# Brain MRI in infants exposed to the Zika virus, with one-year follow-up: expanding the phenotype

**DOI:** 10.1590/0100-3984.2024.0014

**Published:** 2024-11-07

**Authors:** Teresa Cristina de Castro Ramos Sarmet dos Santos, Mai-Lan Ho, Maria de Fatima Vasco Aragão, Renata Artimos de Oliveira Vianna, Alexandre Ribeiro Fernandes, Alair Augusto Sarmet Moreira Damas dos Santos, Claudete Aparecida Araújo Cardoso

**Affiliations:** 1 Medical School, Universidade Federal Fluminense (UFF), Niterói, RJ, Brazil; 2 University of Missouri, Columbia, MO, USA; 3 Medical School, Universidade Federal de Pernambuco (UFPE), Recife, PE, Brazil

**Keywords:** Microcephaly, Zika virus infection, Magnetic resonance imaging, White matter/abnormalities, Microcefalia, Infecção por Zika vírus, Ressonância magnética, Substância branca/anormalidades

## Abstract

**Objective:**

To analyze longitudinal changes between two brain magnetic resonance imaging (MRI) exams performed one year apart in symptomatic infants with congenital Zika syndrome (CZS) and normocephalic infants exposed to the Zika virus (ZIKV) prenatally.

**Materials and Methods:**

This was a prospective observational study. Infants born to women who tested positive for ZIKV on reverse transcription-quantitative polymerase chain reaction during pregnancy were classified into two groups: symptomatic infants with CZS and asymptomatic infants. All of the infants underwent brain MRI at presentation and after one year of follow-up. All MRI scans were evaluated independently by a pediatric radiologist and a pediatric neuroradiologist, and the infants underwent clinical monitoring by a pediatric neurologist.

**Results:**

The sample included 36 infants exposed to ZIKV perinatally. Therefore, a total of 72 MRI scans were evaluated. Among the 36 infants included a diagnosis of CZS was made in 25 (69.4%), of whom 18 presented with a combination of classic findings (including reduced brain volume, subcortical calcifications, brainstem hypoplasia, malformations of the corpus callosum, malformations of cortical development, and ventriculomegaly), as well as atypical findings such as hyperintense foci in the white matter on T2-weighted sequences. Of those same 25 infants, seven presented with mild lesions. Of the 11 normocephalic patients, five (13.9%) had atypical findings such as hyperintense foci in the white matter on T2-weighted sequences and no other manifestations of CZS, although there was mild neurological involvement. Six (16.6%) of the 36 patients had completely normal MRI scans with no neurological changes. No disease progression was observed during follow-up.

**Conclusion:**

In infants exposed to ZIKV perinatally, the frequency of classic and atypical findings on brain MRI seems to be associated with the neurological status. Brain MRI is an important diagnostic tool in the evaluation and monitoring of patients with congenital infection, because intracranial changes other than microcephaly can occur.

## Abstract (pt)

**Objetivo:**

Analisar as alterações longitudinais entre dois exames realizados com intervalo de um ano de ressonância magnética (RM) cerebral em bebês sintomáticos com síndrome congênita pelo ZIKA vírus (SCZ) e normocefálicos com exposição prenatal ao Zika vírus (ZIKV).

**Materiais e Métodos:**

Este é um estudo observacional prospectivo. Os bebês expostos ao ZIKV durante a gestação foram classificados em dois grupos: crianças sintomáticas com SCZ e crianças sem SCZ com PCR-RT materno positivo. Todos os participantes foram submetidos a exames de RM cerebral na apresentação e após um ano de acompanhamento, com avaliação independente por um radiologista pediátrico e um neurorradiologista pediátrico, além do acompanhmento clínico por neurologista pediátrico.

**Resultados:**

A amostra incluiu 36 bebês com exposição perinatal ao ZIKV e a análise de 72 exames de RM. Desses 36 bebês, 25 (69,4%) apresentavam SCZ e 18 desses 25 com combinação de achados clássicos (volume cerebral reduzido, calcificações subcorticais, hipoplasia do tronco cerebral, malformações do corpo caloso e do desenvolvimento cortical, ventriculomegalia e outros) e achados atípicos como focos hiperintensos no T2 na substância branca, sendo 7/25 com lesões discretas. Dos 11 pacientes normocefálicos, 5/36 (13,9%) apresentavam achados atípicos como focos hiperintensos no T2 na substância branca e sem outras manifestações da SCZ, porém afetados leve neurologicamente, e um 6/36 (16,6%) casos com RM completamente normal, sem alteração neurológica. Nenhuma progressão da doença foi observada durante o acompanhamento.

**Conclusão:**

A frequência de achados clássicos e atípicos na RM na infecção congênita por ZIKV está associada ao estado neurológico. A RM é uma ferramenta diagnóstica importante na avaliação e no acompanhamento de pacientes com infecção congênita, pois outros achados intracranianos podem ocorrer além da microcefalia.

## INTRODUCTION

Zika virus (ZIKV) is a mosquito-borne flavivirus that has been endemic in some tropical countries for many years, with mild disease outbreaks reported sporadically. In 2015, a large outbreak of ZIKV infection with severe disease manifestations led the World Health Organization (WHO) to declare a public health emergency^(1)^. In particular, the association of prenatal ZIKV infection with fetal and neonatal microcephaly^(2)^ prompted the use of noninvasive imaging to characterize associated central nervous system (CNS) abnormalities^(3-6)^.

In recent years, several neuroimaging studies have reported abnormalities typical of ZIKV infection. However, there are a number of confounding factors, including small study populations, limited follow-up, the use of different imaging modalities^(3-7)^, and the inclusion of normocephalic individuals^(7,8)^. Over time, researchers have established that microcephaly is only one of several neurologic and developmental abnormalities associated with intrauterine exposure to ZIKV^(7-9)^, especially if the infection occurs in the first or early second trimester of pregnancy. These abnormalities are collectively referred to as congenital Zika syndrome (CZS).

Laboratory confirmation of ZIKV infection during the brief period of maternal viremia is technically challenging, especially in pregnant women who are asymptomatic. Clinical and radiological biomarkers become critical for the diagnosis of CZS after the window for maternal laboratory diagnosis has passed. Magnetic resonance imaging (MRI) offers a noninvasive diagnostic tool to complement the clinical neurological assessment of infants exposed to ZIKV *in utero*, those with microcephaly as well as those who are less affected (normocephalic). The aim of this study was to characterize abnormalities on brain MRI over time in a broad spectrum of infants exposed to ZIKV perinatally, correlating the imaging findings with the clinical and neurological aspects.

## MATERIALS AND METHODS

This was a prospective observational study conducted at the Pediatric Infectious Diseases Clinic and the Radiology Department of Antônio Pedro University Hospital, operated by Fluminense Federal University, and at the Imaging Center of the Niterói Hospital Complex. The study was conducted in accordance with the Declaration of Helsinki and approved by the research ethics committees of the respective institutions (Reference no. 62995516.8.0000.5243). Infants exposed to ZIKV during gestation were referred to the Clinical Research Unit of the hospital from other health care facilities in the metropolitan area of the city of Niterói, located in the Brazilian state of Rio de Janeiro. Written informed consent was obtained from the mothers or legal guardians of the newborns and infants. Infants were enrolled from November 2015 to August 2018 and followed clinically up to five years of age. Study reporting guidelines followed the Strengthening the Reporting of Observational Studies in Epidemiology guidelines for observational studies^(10)^. Data generated or analyzed during the study are available from the corresponding author upon request.

### Participants

The sample of infants included two groups: symptomatic infants with CZS confirmed or not by RNA amplification with reverse transcription-quantitative polymerase chain reaction (RT-qPCR) of maternal body fluids; and asymptomatic infants exposed to ZIKV during pregnancy, confirmed by maternal RT-qPCR. Infants with perinatal hypoxic-ischemic injury were excluded, as were those with serologically confirmed syphilis, toxoplasmosis, rubella, cytomegalovirus (CMV) infection, herpes infection, or nonprogressive encephalopathy from other causes, as well as those in whom maternal RT-qPCR was not performed and the results of the first MRI and neurological examinations were normal.

### Clinical definitions

Gestational age (GA) at birth, in weeks, was determined based on date of the last menstrual period and obstetric evaluation. The Z-scores for birth weight and head circumference (HC) were classified according to the criteria of the International Fetal and Newborn Growth Consortium for the 21st Century (INTERGROWTH-21st) Project^(11)^. Small-for-GA infants were defined as those with a birth weight below the 10th percentile for GA. According to WHO criteria, microcephaly at birth was characterized by HC measurement within 24 h after birth and confirmed within the first week of life using a standardized technique and mean INTERGROWTH-21st Project metrics. Microcephaly was confirmed when the HC was less than two standard deviations below the mean for sex and GA^(12)^. That standard was applied to infants born at term, with mean HC cutoffs of 31.9 cm for male infants and 31.5 cm for female infants^(13)^, as well as to preterm (< 37 weeks GA) infants.

All of the infants in our sample were followed by a clinical team consisting of a pediatrician, a pediatric infectious disease specialist, and pediatric neurologists, who ordered MRI examinations based on screening imaging (ultrasound or computed tomography of the head) and neurological examination. All of the infants underwent neurological assessment with two neurodevelopmental scales: the WHO Gross Motor Development Assessment^(13)^; and the Denver Developmental Screening Test II (DDST-II) for language, psychosocial, gross motor, and fine/adaptive motor development^(14)^. Infants underwent neurological examination by two pediatric neurologists at enrolment, as well as at 4, 8, 12, 18, and 24 months of age. For the purposes of this study, patients were separated into neurologically affected and unaffected patients. Affected patients were classified as follows: mild, when the infant presented with delays on the DDST-II; moderate, when the infant presented with delays on the DDST-II and motor abnormalities such as hypertonia, hypotonia, spasticity, dysphagia, dyskinesia, and dystonia; or severe, when the infant presented with delays on the DDST-II, motor abnormalities, and epilepsy based on the International League Against Epilepsy classification^(15)^.

### Laboratory tests

The ZIKV infection was confirmed in the mothers of infants exposed to the virus with detectable RNA in blood and/or urine samples^(9)^. All RT-qPCR assays were performed at a referral laboratory for the diagnosis of arboviruses, and the results were confirmed by the Fluminense Federal University Multiuser Laboratory for Research Support in Nephrology and Medical Sciences. Blood and urine samples collected from pregnant women during the acute infection (rash) period were tested for arbovirus in accordance with the Brazilian guidelines^(16)^: up to five days after rash onset in serum; and up to 14 days after rash onset in urine. In addition, RT-qPCR was performed on samples collected from all infants with a clinical diagnosis of microcephaly at birth.

The main congenital infectious diseases (toxoplasmosis, CMV infection, and rubella), syphilis, and HIV infection were excluded through specific laboratory tests. In all cases, tests were performed in the Clinical Pathology Department or test results were obtained from prenatal reports.

### MRI protocols

All patients underwent a standardized imaging protocol, which included an axial rapid T2-weighted localizer sequence, with a repetition time/echo time (TR/TE) of 4,330/104 ms, to prescribe the subsequent scan coverage. The initial MRI sequences were acquired in a 1.5-T scanner (Symphony; Siemens, Erlangen, Germany) with a sagittal three-dimensional T1-weighted sagittal sequence (TR/TE, 522/14 ms); axial and coronal turbo spin-echo T2-weighted sequences (TR/TE, 4,000/99 ms); axial fluidattenuated inversion recovery (FLAIR, TR/TE/inversion time, 9,000/114/2,500 ms); and diffusion-weighted imaging (DWI) and susceptibility-weighted imaging (SWI) without intravenous gadolinium. Because of equipment updates, the MRI examinations at one year of follow-up MRI were performed in a 3.0-T scanner (Ingenia; Phillips Medical Systems, Eindhoven, the Netherlands), including a three-dimensional T1-weighted sequence, a two-dimensional turbo spin-echo T2-weighted sequence, a FLAIR sequence, DWI, and SWI, with similar TR/TE parameters employed in the initial MRI sequences.

All MRI scans were randomized and reviewed independently by a senior pediatric radiologist (with 20 years of experience) and a senior pediatric neuroradiologist (with 32 years of experience), both of whom were blinded to the identity, sex, and date of birth of the infant, as well as to the GA, HC, maternal history of skin rash, maternal RTqPCR results, and neurological findings. Disagreements were resolved by consensus. Images were evaluated for the following^(3-8,17-23)^: brain volume loss; enlarged cerebrospinal fluid spaces; calcifications in the subcortical white matter, cerebellar white matter, basal ganglia, and brainstem; microcephaly; brainstem hypoplasia; cerebellar hypoplasia/ atrophy; dysgenesis/agenesis of the corpus callosum; mega cisterna magna; malformations of cortical development; ventriculomegaly; and periventricular white matter hyperintense foci on T2-weighted and FLAIR sequences. All data were correlated with GA at infection and neurological clinical data.

### Statistical analysis

To compare the frequencies of various brain MRI findings between the infants with (mild, moderate, or severe) neurological manifestations and the unaffected infants, we used Fisher’s exact test. Subject-wise two-tailed paired t-tests were employed to compare the frequencies of findings between the two time points. Results are expressed as absolute and relative frequencies or as medians and interquartile ranges (IQRs). Values of *p* < 0.05 were considered statistically significant. Data were analyzed with R software, version 3.6.1 (R Foundation for Statistical Computing, Vienna, Austria).

## RESULTS

### Demographics and summary findings

**Figure 1:**
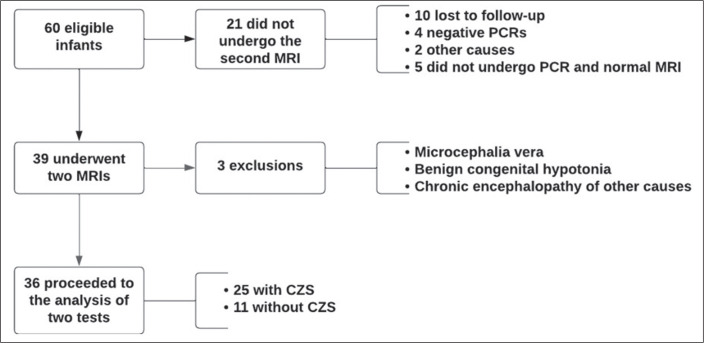
Sample selection process.

A total of 246 infants exposed to ZIKV were enrolled in our clinical cohort, which was evaluated in a previous study^(17)^. In the present study, our initial sample comprised 60 infants who were referred for MRI during 2017. Of those, 37 (61.7%) were male. The median age at the initial MRI scan was 12 months (IQR, 8.0-13.5 months). Follow-up MRI scans were acquired approximately 12 months later, during 2018. For various reasons, 21 of the infants did not undergo the follow-up MRI. Consequently, the revised sample comprised 39 infants, with a median age of 24 months (IQR, 21.5-27 months), of whom 27 (69.2%) were male. Following additional exclusions for alternate clinical diagnoses, the final sample consisted of 36 infants who underwent brain MRI in 2017 and 2018 (Figure 1). Among those 36 infants, the maternal RT-qPCR was positive for ZIKV in 20 (55.6%). In the remaining 16 cases, there was no laboratory confirmation of maternal ZIKV infection, for a number of reasons, including the absence of maternal exanthem with specimen collection outside the viremia period, although those cases still met the inclusion criteria (symptomatic infants with CZS).

For all eligible participants, the HC (in cm) was initially measured between 24 h after birth and seven days of life. At that time, the mean HC was 32 cm (IQR, 29.8-34 cm). Of the 36 infants evaluated, 25 (69.4%) presented with microcephaly at birth and in the initial postnatal period. Of those 25, four (one with CZS) subsequently showed normalization of HC at the one-year follow-up examination. Eleven infants (30.6%) were normocephalic at birth.

**Figure 2:**
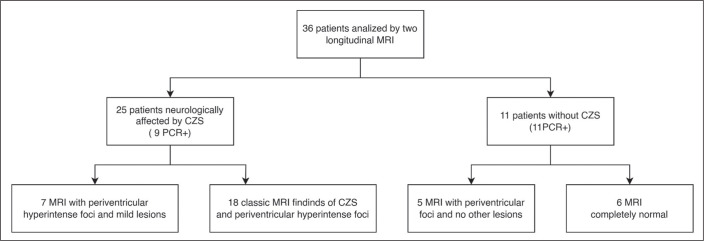
Clinical and radiological classification of 36 cases in infants exposed to ZIKV perinatally, based on neurologic features and MRI findings.

Of the 36 infants in our sample, 25 (69.4%) were clinically diagnosed with CZS and 11 (30.6%) had no neurologic abnormalities consistent with CZS, despite a maternal RT-qPCR that was positive for ZIKV. On MRI, 18 (72.0%) of the 25 infants with CZS presented with severe, classic abnormalities and seven (28.0%) presented with milder, nonspecific findings. Of the 11 patients not diagnosed with CZS, five (45.4%) showed periventricular white matter signal abnormalities and were considered by neurologists to be mildly affected, whereas six (54.5%) had normal MRI examinations, with no neurological changes (Figure 2).

### MRI findings

**Figure 3:**
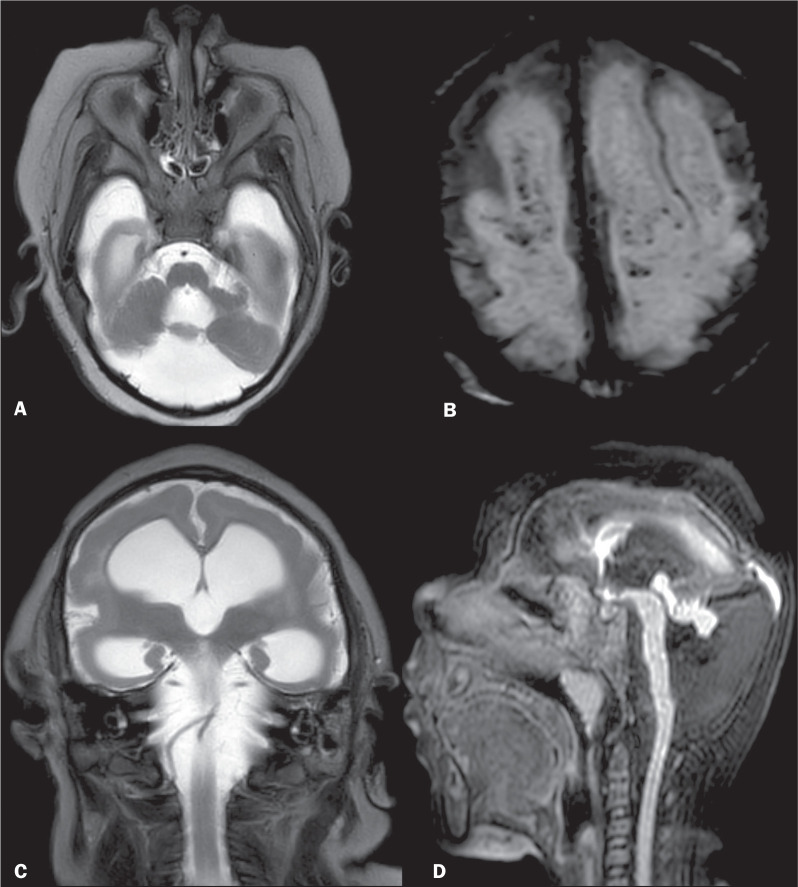
Severe brain injury on MRI scans of different patients. Axial and coronal T2-weighted MRI sequences (A and C, respectively) showing severe global volume loss with cerebellar hypoplasia (A) and ex vacuo enlargement of the lateral ventricles and subarachnoid spaces with pachygyria (C) in a 24-month-old infant. Multifocal punctate calcifications are seen along the gray-white matter junction on SWI sequence (B) in a 13-month-old infant. Sagittal T1-weighted volumetric sequence (D) showing microcephaly, hypogenesis of the corpus callosum, tectal dysplasia, brainstem hypoplasia, cerebellar hypoplasia, and mega cisterna magna in a 9-month-old infant.

**Figure 4:**
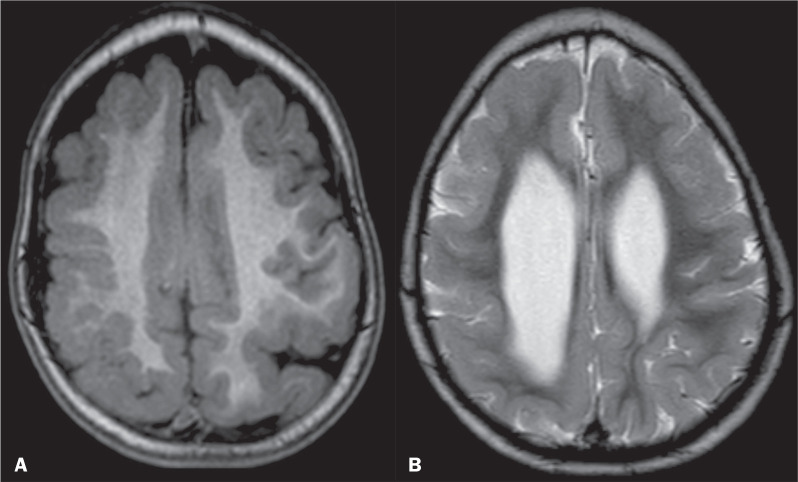
Malformations of cortical development on MRI in a 19-monthold patient. Axial T1-weighted and T2-weighted sequences (A and B, respectively) showing global cerebral volume loss and patchy white matter signal. In the cerebral cortex, note the heterogeneous admixed areas of polymicrogyria and pachygyria.

Neuroimaging findings of CZS, previously described in the literature^(3,4,21-23)^ and recorded in this study, included brain volume loss, which was seen in 18 (50.0%) of the 36 infants and was most severe among the 10 infants (27.8%) in whom maternal infection occurred in the first trimester. All 18 of those patients presented with moderate to severe neurological impairment. Of the 25 infants with microcephaly at birth, 11 (44.0%) had been infected in the first trimester of the pregnancy. Other MRI findings included the following (Figures 3 and 4): subcortical, periventricular, cerebellar, basal ganglia, and brainstem calcifications; brainstem and cerebellar hypoplasia; dysgenesis/ agenesis of the corpus callosum; ventriculomegaly; malformations of cortical development; mega cisterna magna; and enlarged cerebrospinal fluid spaces. All MRI abnormalities were more common among the infants infected in the first trimester, followed by those in whom the timing of infection was undetermined, because the infected mother did not develop a rash. These infants also had neurological symptoms.

**Figure 5:**
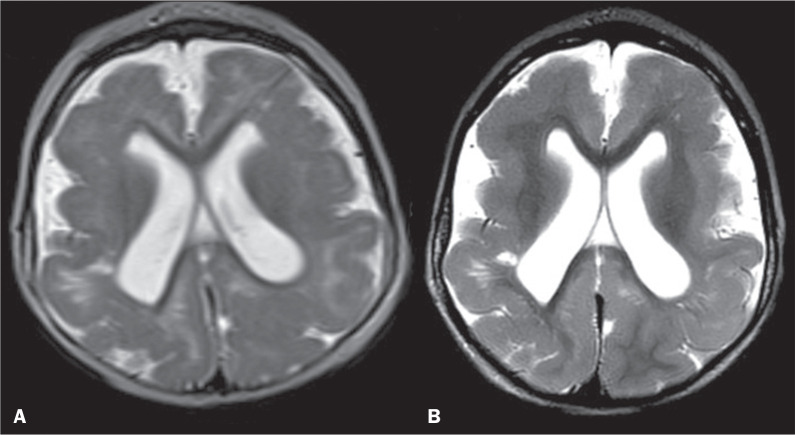
Persistent foci of hyperintensity in the periventricular white matter and areas of pachygyria and polymicrogyria interspersed in the frontal lobes on axial T2-weighted images from the first MRI at 10 months of age (A), similar to the second MRI at 19 months (B).

Less specific neuroimaging findings included abnormal hyperintense signals on T2-weighted and FLAIR sequences and hypointense signals on T1-weighted sequences, in the periventricular white matter, subcortical white matter, or centrum semiovale (Figure 5), which have previously been suggested to represent delayed myelination in congenital ZIKV infection^(19)^. Abnormal signals in the white matter were noted in 30 infants (83.3%), including 18 (50.0%) with severe additional brain malformations. White matter signal changes in the absence of other structural abnormalities were seen on MRI in 12 infants (33.3%), including seven (19.4%) with neurological manifestations of CZS and five (13.9%) with mild neurological changes but without CZS.

There were statistically significant associations between clinical neurological impairment and the classic neuroimaging features of CZS, including brain volume loss, enlarged cerebrospinal fluid spaces, subcortical calcifications, brainstem hypoplasia, cortical malformation, mega cisterna magna, ventriculomegaly, and dysgenesis/ agenesis of the corpus callosum. Nonspecific white matter signal changes were also significantly associated with neurological manifestations (Table 1).

**Table 1:** MRI findings in neurologically affected and unaffected children exposed to ZIKV during the intrauterine period.

Neuroimaging findings	Affected (N = 25) n (%)	Unaffected (N = 11) n (%)	*P*	Total (N = 36) n (%)
Classic findings				
Reduced brain volume	18 (72.0)	0 (0.0)	0.00139	18 (50.0)
Enlarged cerebrospinal fluid spaces	18 (72.0)	0 (0.0)	0.00191	18 (50.0)
Subcortical calcifications	15 (60.0)	0 (0.0)	0.00147	15 (41.7)
Microcephaly at birth/in the postnatal period	25 (100.0)	0 (0.0)		21 (58.3)^*^
Brainstem hypoplasia	11 (44.0)	0 (0.0)	0.03408	11 (30.6)
Cerebellar hypoplasia/atrophy	9 (36.0)	0 (0.0)	0.07602	9 (25.0)
Dysgenesis/agenesis of the corpus callosum	18 (72.0)	2 (18.2)	0.04914	20 (55.6)
Mega cisterna magna	17 (68.0)	0 (0.0)	0.00191	17 (47.2)
Malformation of cortical development	18 (72.0)	0 (0.0)	0.00103	18 (50.0)
Ventriculomegaly	18 (72.0)	0 (0.0)	0.00124	18 (50.0)
Nonspecific findings				
Periventricular hyperintense foci on T2-FLAIR	23 (92.0)	5 (45.4)	< 0.0001	30 (83.3)
Prominence of perivascular spaces	7 (28.0)	4 (36.4)	0.4088	10 (27.8)

T2-FLAIR, T2-weighted and FLAIR (sequences).

* Thirteen patients had microcephaly at birth, and eight patients developed postnatal microcephaly that persisted at the one-year follow-up examination. Four patients (one neurologically affected) had microcephaly at birth that normalized during follow-up. Eleven patients were normocephalic at birth and at the one-year follow-up examination (five neurologically normal; six neurologically affected).

Six patients (16.7%) had normal brain MRI findings and normal neurological development, despite a maternal RT-qPCR confirming prenatal ZIKV exposure. Subjectwise two-tailed paired t-tests did not reveal statistically significant differences between the two time points in terms of the frequencies of MRI abnormalities for individual patients, suggesting that there was no disease progression after one year. There were also no statistically significant differences between the morphological findings described from the initial MRI scans, performed in 1.5-T scanners, and those described from the follow-up MRI scans, performed in 3.0-T scanners.

## DISCUSSION

Our study sample expands the phenotype of prenatal ZIKV infection, including not only the classic presentation of infants with microcephaly and CZS but also that of normocephalic infants with mild or no neurological symptoms, without CZS. We evaluated infants longitudinally, using follow-up brain MRI to assess the presence and severity of anatomical malformations characteristic of ZIKV infection, as well as the possibility of disease progression through imaging over a year. We observed no changes, not even any of the so-called nonspecific white matter changes.

Our study corroborates a systematic review of the literature on neuroimaging findings in congenital ZIKV and its relation to the time of infection^(22)^, which was performed in accordance with the methodology described in the Cochrane Handbook for Systematic Reviews and presented in the Preferred Reporting Items for Systematic Reviews and Meta-Analyses statement. In an initial database search, the authors retrieved 2,214 articles, from which they selected eight key articles describing a collective total of 235 cases of CZS with microcephaly, in which infections occurred in the first trimester of pregnancy with a slight predominance of affected males, as reported elsewhere^(23)^. In the present study, we have described findings in normocephalic infants with later perinatal exposures and milder neurologic symptoms, among whom the frequency and severity of imaging abnormalities was lower overall. For example, loss of brain volume, enlarged cerebrospinal fluid spaces, subcortical calcifications, and ventriculomegaly were present, respectively, in 50.0%, 50.0%, 41.7%, and 50.0% of our sample, which included normocephalic infants who tested positive for ZIKV, compared with 81.5%, 80.0%, 88.2%, and 78.1% of the cases in the previously cited review^(22)^, in which the objective was to study data related to infants with microcephaly.

For abnormalities other than microcephaly, the spectrum of disease varied but the overall proportion of affected infants was similar to that reported in another review of the literature^(21)^, including malformations of cortical development (50.0% vs. 58.5%), mega cisterna magna (47.2% vs. 50.4%), brainstem hypoplasia (30.6% vs. 40.3%), and cerebellar hypoplasia (25.0% vs. 36.1%). We also detected corpus callosum abnormalities in 55.6% of cases, versus 34.4% in that review, as well as white matter changes not described in the systematic review^(22)^, probably due to the exclusive use of MRI in our study, which is more sensitive than are computed tomography and ultrasound for mild malformations^(24)^, which were also included in the aforementioned review^(22)^.

Our study is important and unique for its longitudinal follow-up of infants infected with ZIKV using MRI, in a five-year cohort monitored by a multidisciplinary team. Using one-year follow-up MRI data allowed us to confirm the absence of disease progression. Paired t-tests failed to reveal statistically significant changes in the frequencies of imaging abnormalities between serial examinations. When reviewing temporal changes on a case-by-case basis, we found that, in some infants, there was a change in clinical classification between borderline microcephaly and microcephaly. Given that normal HC values vary with age, these changes likely relate to physiologic changes in growth, cerebrospinal fluid circulation, and the extrauterine environment. Despite the head growth, severity scores for brain atrophy and enlarged cerebrospinal fluid spaces were relatively stable over time (none/mild vs. moderate/severe). Features that are less common, such as pontocerebellar and corpus callosum hypoplasia, were consistently identified on both MRI examinations. For some infants with subtle white matter abnormalities or calcifications, progressive myelination at the later time point made signal changes in the white matter more apparent. Nevertheless, a retrospective review of these cases showed that abnormalities could be detected at both time points on multiple sequences, including T1weighted and SWI sequences.

Van der Voorn et al.^(25)^ compared children with congenital CMV infection and abnormal white matter signal, children with periventricular leukomalacia (PVL) from other causes, and children without a neuropathological diagnosis (controls). No significant differences were found between the two pathologies, suggesting that the neuropathological substrate of white matter injury in congenital CMV infection and PVL follow a common final pathway with developmental injury to immature oligodendrocytes^(2,25,26)^.

It has been demonstrated that ZIKV shows tropism for the CNS; early infection of fetal neuronal cells is thought to inhibit neuronal cell differentiation and promote inflammatory mediators, leading to delayed brain growth and reduced neuronal viability^(27)^. This results in a spectrum of congenital brain malformations depending on the timing and severity of infection. Nonspecific white matter signal abnormalities have also been reported in ZIKV infection, which, according to hypotheses raised in the articles consulted, could be related to delayed myelination^(19)^ or myelinoclastic lesions^(28)^. Given that the lesions did not progress during follow-up in our sample, as determined by the pediatric neuroradiology specialists who analyzed myelination in relation to that expected for age, they were probably myelinoclastic lesions. Those lesions could be analogous to the white matter lesions seen in CMV infection and PVL, corresponding pathologically to the destruction of axonal myelin after oligodendrocyte damage and astrogliosis^(25-28)^. Like CMV, ZIKV inhibits the induced differentiation of precursor cells into neurons and may have similar effects on differentiation in other cells, such as macrophages and dendritic cells^(2)^. In one postmortem neuropathological study of fetuses with classic neuroimaging findings^(29)^, the authors identified three patterns of lesions: one in which ventriculomegaly was severe because of midbrain injury with stenosis/distortion of the aqueduct; one in which there was a reduction in brain volume with mild/moderate ventriculomegaly; and one in which the brain was well formed with sparse calcifications, coinciding with late infection, although with loss of descending fibers that resulted in hypoplasia of the pons, pyramids, and corticospinal tracts. Loss of spinal motor cells explained the intrauterine akinesia, arthrogryposis, and neurogenic muscular atrophy^(29)^. The virus may also disrupt oligodendrocyte maturation, leading to axonal development failure and, ultimately, axonal degeneration^(30,31)^. In addition, changes in the microenvironment of the developing brain, due to cytokines generated by glial cells and infiltrating immune cells, can induce cell apoptosis in the fetal brain^(2,30,31)^ resulting in multifactorial white matter injury. That hypothesis is further supported by our finding of the persistence and occasionally increased visibility of white matter lesions demonstrating hypointense signals on T1-weighted imaging and mineralization against a background of progressive brain maturation and myelination.

The limitations of our study include the small sample size, the potential for underdiagnosis due to various clinical factors, and the failure to include automated methodologies for assessing brain volume (e.g., FreeSurfer), as well as the single-center follow-up with associated patient attrition and MRI equipment considerations. Nevertheless, we were able to confirm a spectrum of MRI findings in the sample at initial presentation and at one year of follow-up and to correlate those findings with the severity of neurological symptoms. We have confirmed a range of anatomic brain findings in infants with CZS, which also manifested to a lesser extent in affected normocephalic infants with prenatal ZIKV exposure. Therefore, brain MRI is a useful noninvasive diagnostic tool that facilitates the initial evaluation and follow-up assessment of infants exposed to ZIKV during pregnancy, aiding clinicians in the diagnostic and prognostic assessment of such patients.

## CONCLUSION

In congenital ZIKV infection, the frequency of classic and atypical findings on brain MRI seem to be associated with the neurological status. Infants with proven exposure due to maternal ZIKV infection during pregnancy may present hyperintense lesions in periventricular white matter on T2-weighted and FLAIR sequences, a finding present in 83.3% of our sample. Therefore, a normal HC without serious clinical abnormalities might not indicate the absence of CNS involvement. Interestingly, these findings are also similar to those found in the literature on congenital CMV infection, suggesting that white matter lesions in congenital infection with CMV or ZIKV are similar to those found in PVL and may be due to damage and loss of oligodendrocytes in the developing white matter. Brain MRI is an important diagnostic tool in the assessment and follow-up of patients with congenital infection, because intracranial changes other than microcephaly can occur.
